# 195. Comparative Analysis Between Brucellosis Cases With And Without Liver Involvement

**DOI:** 10.1093/ofid/ofad500.268

**Published:** 2023-11-27

**Authors:** Fatma Hammami, Makram Koubaa, Khaoula Rekik, Amal Chakroun, Fatma Smaoui, Chakib Marrakchi, Mounir Ben Jemaa

**Affiliations:** Infectious Diseases Department, Hedi Chaker University Hospital, University of Sfax, Tunisia, Sfax, Sfax, Tunisia; Infectious Diseases Department, Hedi Chaker University Hospital, University of Sfax, Tunisia, Sfax, Sfax, Tunisia; Infectious Diseases Department, Hedi Chaker University Hospital, University of Sfax, Tunisia, Sfax, Sfax, Tunisia; Infectious Diseases Department, Hedi Chaker University Hospital, University of Sfax, Tunisia, Sfax, Sfax, Tunisia; Infectious Diseases Department, Hedi Chaker University Hospital, University of Sfax, Tunisia, Sfax, Sfax, Tunisia; Infectious Diseases Department, Hedi Chaker University Hospital, University of Sfax, Tunisia, Sfax, Sfax, Tunisia; Infectious Diseases Department, Hedi Chaker University Hospital, University of Sfax, Tunisia, Sfax, Sfax, Tunisia

## Abstract

**Background:**

Brucellosis, a multisystem disease, involve mostly the reticuloendothelial system, but it can also affect any other organ. Liver involvement is often present during the acute phase. We aimed to compare clinical and laboratory features of brucellosis cases with and without liver involvement.

**Methods:**

We conducted a retrospective study including all acute brucellosis cases hospitalized in the infectious diseases department between 1992 and 2022. Liver involvement were defined as a serum alanine transaminase > 40 U/L and/or a serum aspartate aminotransferase > 40 U/L.

**Results:**

We encountered 105 cases, among which 46 had liver involvement (43.8%). A male predominance was noted among cases with (69.6%) and without (61%) liver involvement, with no significant difference (p=0.363). Vomiting (19.6% vs 5.1% ; p=0.021), nausea (28.3% vs 3.4% ; p< 0.001) and loss of appetite (34.8% vs 10.2% ; p=0.002) were significantly more frequent among cases with liver involvement. Fever (89.1% vs 89.8% ; p=1), arthralgia (52.2% vs 61% ; p=0.364) and myalgia (37% vs 33.9% ; p=0.745) were noted among cases with and without liver involvement. Blood culture positivity was significantly more frequent among cases with liver involvement (43.5% vs 5.1% ; p< 0.001). An elevated C-reactive protein level (85.7% vs 28.8% ; p=0.002), an accelerated erythrocyte sedimentation rate (76.1% vs 54.2% ; p=0.021) and thrombocytopenia (39.1% vs 13.6% ; p=0.003) were significantly more frequent among cases with liver involvement. Anemia (39.1% vs 25.4% ; p=0.133) and leukopenia (21.7% vs 22% ; p=0.971) were noted among cases with and without liver involvement, with no significant difference.

**Conclusion:**

Acute brucellosis cases with liver involvement were characterised by frequent vomiting, nausea and loss of appetite as clinical symptoms, besides elevated inflamatory markers and frequent thrombocytopenia, which might be explained by the involvement of the reticuloendothelial system.

**Disclosures:**

**All Authors**: No reported disclosures

